# Recurrent Embolic Strokes of Undetermined Source in a Patient with Extreme Lipoprotein(a) Levels

**DOI:** 10.3389/fneur.2016.00144

**Published:** 2016-08-31

**Authors:** Zachary Bulwa, Audrey Kim, Karandeep Singh, Alexander Kantorovich, Faten Suhail

**Affiliations:** ^1^Internal Medicine, University of Chicago – NorthShore University Health System, Evanston, IL, USA; ^2^Rosalind Franklin University of Medicine and Science, North Chicago, IL, USA; ^3^College of Pharmacy, Chicago State University, Chicago, IL, USA; ^4^Internal Medicine, Advocate Christ Medical Center, Chicago, IL, USA

**Keywords:** stroke, embolic stroke of undetermined source, cryptogenic stroke, lipoprotein(a), carotid atherosclerosis, carotid stenting

## Abstract

Lipoprotein(a) is a plasma lipoprotein and known cardiovascular risk factor, most recently implicated in the development of high-risk carotid atherosclerotic plaques without significant carotid stenosis. We present a case of a young African-American female with recurrent embolic strokes of undetermined source. After our thorough investigation, we identified the link between a small, irregular plaque in the right internal carotid artery, and an extremely elevated plasma level of lipoprotein(a) as the source of her embolic strokes.

## Case Report

We report a case of a 46-year-old female with a past medical history significant for multiple right-sided cerebral ischemic events who was transferred from an outside hospital with acute left upper and lower extremity weakness. At the outside hospital, computed tomography (CT) imaging demonstrated an acute middle cerebral artery infarct. Due to contraindications, she did not receive tissue plasminogen activator (tPA).

**Figure 1:**
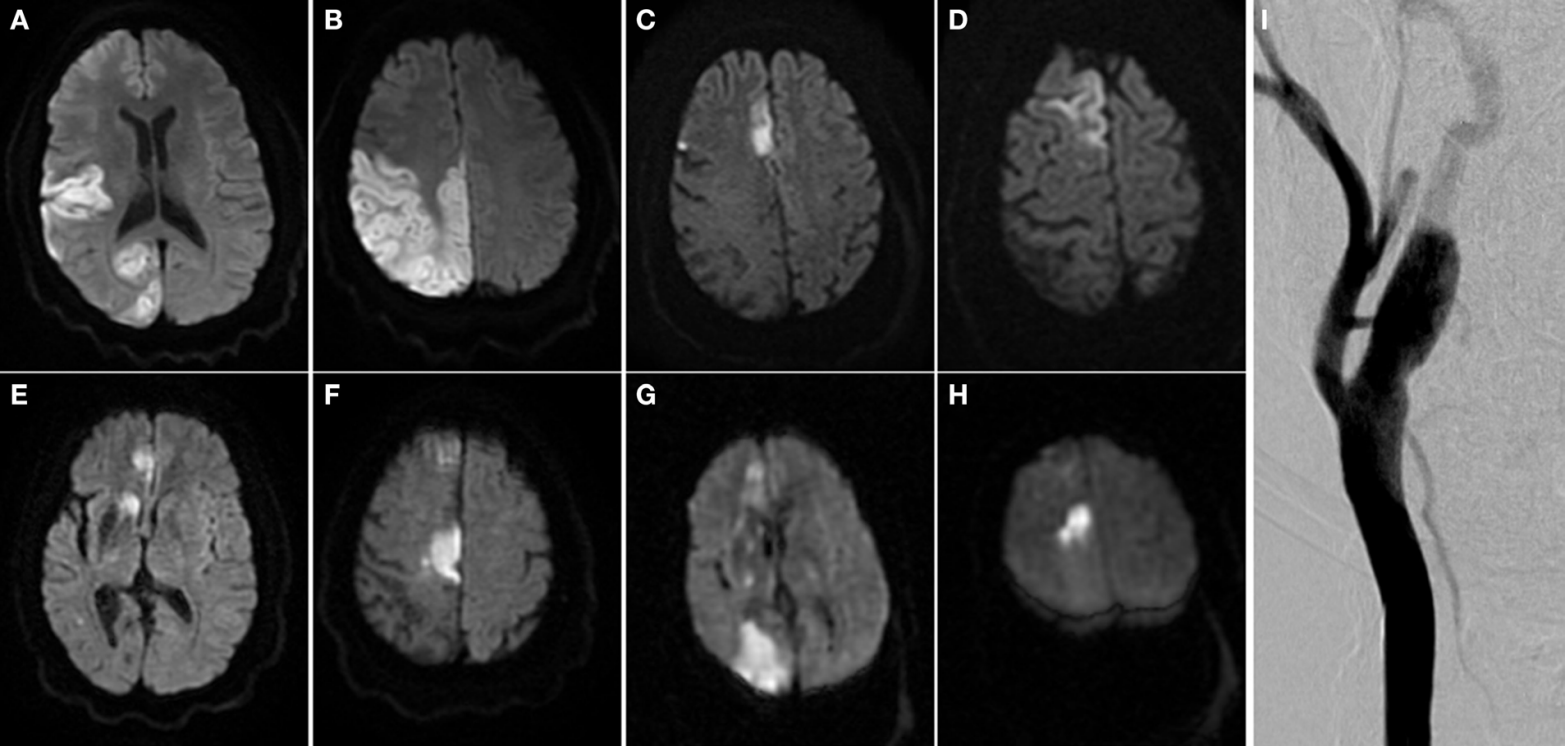
**Multiple embolic territory strokes secondary to irregular and vulnerable carotid atherosclerotic plaque**. **(A–H)** Brain MRI scans from multiple ischemic events over multiple admissions. **(I)** Right carotid angiogram. Arrow indicates atherosclerotic plaque in right internal carotid artery.

Upon transfer, magnetic resonance imaging (MRI) and magnetic resonance angiography (MRA) demonstrated occlusions in the right anterior cerebral artery and middle cerebral artery, and a focal, irregular plaque in the right internal carotid artery (ICA). Cerebral angiogram redemonstrated the plaque, but no immediate intervention was recommended due to non-hemodynamically significant stenosis of less than 20% (Figure 1).

The patient’s vitals, complete blood count, comprehensive metabolic panel, coagulation studies, and lipid panel were all within normal limits upon admission. The patient was placed on continuous cardiac telemetry. Secondary prevention was optimized by the addition of clopidogrel to the standard secondary prevention she was already receiving prior to admission, which included aspirin and atorvastatin.

One week later, the patient complained of a new onset headache. With no history of headache, precautions were taken, and a repeat MRI revealed a new infarct in the right occipital lobe and frontal lobe.

Electrocardiographic, echocardiographic (including transesophageal echocardiogram), and neuroimaging studies exhibited no major-risk cardioembolic source. Hypercoagulability workup was negative except for an extremely elevated plasma lipoprotein(a) level (414 mg/dL). The patient underwent stent placement to stabilize her vulnerable ICA plaque. She was then discharged to an acute rehabilitation facility 2 days later in stable condition.

## Discussion

We describe a young African-American female who meets criteria for recurrent embolic strokes of undetermined source (ESUS) (1). After thorough diagnostic investigation, as outlined by Nouh et al. (2), two pertinent abnormalities were found: a small, irregular plaque in the right ICA and an extremely elevated plasma level of lipoprotein(a) [Lp(a)].

Lipoprotein(a) is a known cardiovascular risk factor, but controversy surrounds its utility as a screening tool and our understanding of its pathological mechanisms (3). Lipoprotein(a) is a plasma lipoprotein consisting of a cholesterol-rich low-density lipoprotein (LDL) particle with one molecule of apolipoprotein B100 covalently linked *via* a disulfide bond to apolipoprotein(a) (3, 4). Plasma levels of Lp(a) are directly related to the variable copy number of kringle IV type 2 (KIV-2) repeats of the apolipoprotein(a) isoforms in the *LPA* gene (3, 4). Smaller isoforms of apolipoprotein(a) are associated with higher concentrations of Lp(a) (3, 5, 6). Lipoprotein(a) levels vary widely in humans, with up to 90% genetically determined *via* the *LPA* gene (6, 7). Individuals with smaller apolipoprotein(a) isoforms have roughly a twofold higher risk of ischemic stroke and coronary heart disease (5). Thus, there is a causal link evidenced by Mendelian randomization between genetically determined elevated levels of Lp(a) and cardiovascular disease (3–5).

Two aspects of Lp(a) make it exceptionally interesting: first, the apolipoprotein(a) co-factor is structurally homologous to plasminogen, but without fibrinolytic activity; and second, the metabolism and distribution of Lp(a) differs from that of LDL (8, 9). Nevertheless, the physiologic function of Lp(a) remains elusive, although its atherogenicity is thought to be due to the interference of the fibrinolytic pathway among other interactions (8).

Cerebral vascular disease, peripheral vascular disease, and most recently, carotid atherosclerosis have been associated with elevated serum levels of Lp(a) (8). These associations are independent of the levels of cholesterol and other traditional risk factors, such as age, gender, smoking, systolic blood pressure, antihypertensive medication use, diabetes mellitus, LDL cholesterol, HDL cholesterol, and triglycerides (10–12).

Virani et al. contend that elevated Lp(a) in African-Americans may confer a higher risk for a cerebrovascular event than for a coronary vascular event (13). They found a positive correlation between Lp(a) levels and ischemic stroke at plasma levels greater than 30 mg/dL in the African-American population (12, 13). Most recently, Zhao et al. identified that elevated Lp(a) levels were associated with high-risk carotid atherosclerotic plaque features. Although these high-risk plaques were often seen in less severe carotid stenosis, they were vulnerable to embolization and were associated with an increased rate of strokes (14). In the ongoing CAPIAS study, which hopes to provide further insight into the role of non-stenosing carotid artery plaques, nearly half of the initially enrolled patients were found to have cryptogenic stroke as defined by TOAST criteria (15, 16).

The extreme plasma level of Lp(a) in combination with an irregular carotid plaque offered few therapeutic options for optimizing secondary prevention in the setting of recurrent ESUS. The promise Lp(a) holds is that, as an independent risk factor for cardiovascular disease, reducing elevated plasma levels will directly reduce cerebrovascular disease (1, 17). A promising new class of lipid-lowering drugs are the proprotein convertase subtilisin/kexin type 9 (PCSK9) inhibitors. PCSK9 regulates the catabolism of Lp(a) by inhibiting Lp(a) internalization *via* the LDL receptor (18). In a pooled analysis of phase II trials, PCSK9 inhibition (*via* evolocumab) resulted in statistically significant dose-related reductions in Lp(a), although no results have indicated a reduction of cerebrovascular events (19). And more recently, PCSK9 has been found complexed to Lp(a) particles in humans with elevated Lp(a) levels (20). It remains to be determined whether lowering Lp(a) levels directly will result in lower cardiovascular event rates, which may provide an additional therapeutic mechanism: antisense oligonucleotides directed against apo(a) (17). Possibly, the most compelling evidence comes from the Pro(a)Life Study Group in which chronic lipoprotein apheresis was shown to reduce the risk of recurrent cardiovascular events (and the need for carotid stenting) over a 2-year period in patients on maximally tolerated lipid-lowering therapy (21).

While long-term consideration of our patient’s chronically elevated plasma Lp(a) level is critical, acute stabilization of the irregular and vulnerable carotid plaque was deemed most important due to the rapid timeline of embolic stroke recurrence. The patient was on aspirin upon admission, but no anticoagulation, consistent with recommendations from the WARSS trial (22). Although there is evidence in a subgroup analysis that warfarin may be more efficacious than aspirin among patients with cryptogenic stroke who did not have hypertension and did have embolic infarcts on neuroimaging, we did not initiate anticoagulation due to concern for an increase in major hemorrhages (22, 23). Currently, two trials are underway to investigate the benefit and risk of anticoagulation with direct oral anticoagulants (DOACs) compared with aspirin in patients with embolic stroke of undetermined source (24, 25). Due to the lack of currently available therapeutic options and probable association to the recurrent strokes, a decision was made to stabilize the carotid clot *via* stenting.

## Conclusion

Considering the clinical picture, imaging, and accompanying laboratory data, it is probable that the irregular and vulnerable carotid atherosclerotic plaque contributed to recurrent ESUS. Thus, it is likely that Lp(a), in contributing to the development of the plaque, indirectly triggered these embolic infarctions. We suggest measuring Lp(a) in the workup of recurrent embolic stroke of undetermined source in the setting of non-hemodynamically significant carotid stenosis in young African-American individuals. Further study will hopefully determine the pathogenic mechanism of elevated Lp(a) plasma levels in order to optimize current therapies and yield new potential therapeutic targets. The recent introduction of PCSK9 inhibitors offers clinicians a novel therapeutic approach to reduce Lp(a) levels, but more research needs to be undertaken to evaluate cerebrovascular outcomes. Results from current randomized controlled trials in patients with ESUS will ideally illustrate the best therapeutic options in the interim (24, 25).

## Ethics Approval

Our case report is exempt from the ethics review process as it does not involve research subjects but rather data available due to a permission form signed by the patient. This consent form allows for the publication of material relating to them in scientific and medical journals. The patient was competent and capable when signing this form.

## Author Contributions

All of the authors participated in the treatment of the patient. ZB was primarily in charge of the patient’s care, while FS was the attending physician. A thorough review of the literature as well as the first draft of the manuscript was completed by ZB. All authors listed, have made substantial, direct and intellectual contribution to the work, and approved it for publication.

## Conflict of Interest Statement

The authors declare that the research was conducted in the absence of any commercial or financial relationships that could be construed as a potential conflict of interest.
